# L'ostéochondrite disséquante du capitellum chez l'adolescent: à propos d'un cas et revue de la littérature

**DOI:** 10.11604/pamj.2014.17.128.3848

**Published:** 2014-02-24

**Authors:** Moncef Erraji, Abdessamad Kharraji, Najib Abbassi, Abdeljawad Najib, Hicham Yacoubi

**Affiliations:** 1 Unité de Chirurgie Orthopédique et Traumatologique, Centre Hospitalier Universitaire d'Oujda, Oujda, Maroc

**Keywords:** osteochondrite disséquante, capitellum, coude, Osteochondritis dissecans, capitellum, elbow

## Abstract

L'osteochondrite disséquante du capitellum est une lésion rare. Elle est généralement attribuée à des microtraumatismes répétés ou à un accident ischémique. Nous en rapportons un cas chez un adolescent tennisman, traité chirurgicalement.

## Introduction

L'ost éochondrite disséquante du capitellum est une pathologie peu fréquente. Toutefois, chez certains sportifs de haut niveau tels que les gymnastes et les adolescents pratiquant des sports de lancer comme le tennis, l'incidence est plus élevée [1]. Le traitement reste discuté, allant du traitement conservateur aux différentes techniques de greffe, en passant par le simple curetage [2, 3]. Nous rapportant un cas d'osteochondrite du capitellum, chez un adolescent sportif (tennis), traitée chirurgicalement.

## Patient et observation

Il s'agit d'un adolescent âgé de 16 ans, sportif (tennis) qui se plaignait depuis plusieurs mois de douleurs mécaniques du coude droit. La symptomatologie s’était aggravée, une semaine avant sa consultation, suite à une chute sur la paume de la main droite, coude en valgus et légère extension. Il se présentait avec une impotence fonctionnelle totale du coude droit. L'examen clinique mettait en évidence une attitude de traumatisé du membre supérieur, avec un oedème du coude associé à une prono-supination limitée par la douleur.

**Figure 1:**
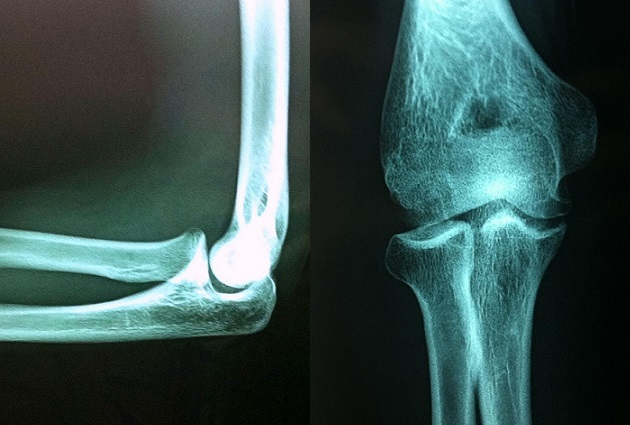
Foyer de condensation sous-chondral du capitellum: objectivé à la Radiographie du coude droit

La radiographie du coude droit objectivait un foyer de condensation sous chondral avec une ossification irrégulière de la tête radiale faisant un stade 5 selon la classification de Baumgarten (Figure 1).

**Figure 2:**
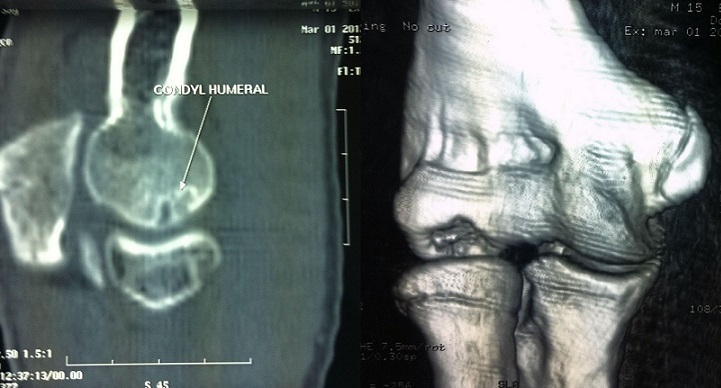
Aspect scannographique du coude droit: localisation antéro-inférieure, niche vide

La tomodensitométrie du coude mettait en évidence des images de defect avec aplatissement de la région sous-chondrale du capitellum huméral. Ce défect est entouré d'une ostéosclérose périphérique mesurant 14 mm de grand axe (Figure 2).

**Figure 3:**
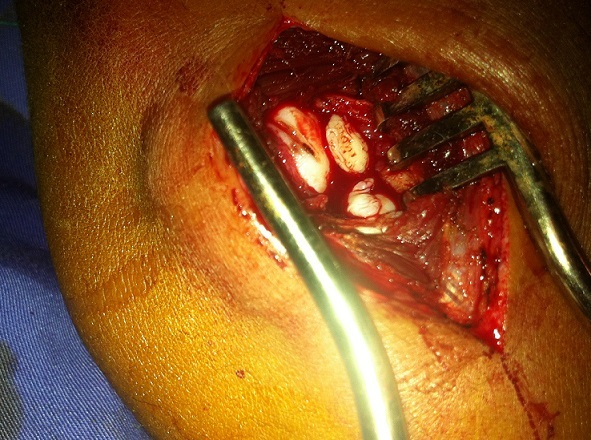
Image per opératoire montrant deux corps étrangers cartilagineux libres en intra-articulaire

Le traitement a été chirurgical à ciel ouvert par voie d'abord antérolatérale classique avec conservation du ligament collatéral latéral et du ligament annulaire du coude. Nous avons réalisé un avivement de la lésion du capitellum et une extraction de deux corps étrangers cartilagineux libres en intra-articulaire, mesurant respectivement 10 mm et 4 mm de grand axe. Des micros fractures ont été réalisées pour stimuler la formation d'un fibrocartilage (Figure 3).

Une rééducation a été débutée, dès l'amélioration des phénomènes inflammatoires, par une auto mobilisation passive, puis mobilisation active et passive douce sans résistance jusqu'au troisième mois postopératoire. à partir du quatrième mois on a institué un travail proprioceptif en chaîne ouverte et fermée, ainsi qu'un renforcement musculaire.

**Figure 4:**
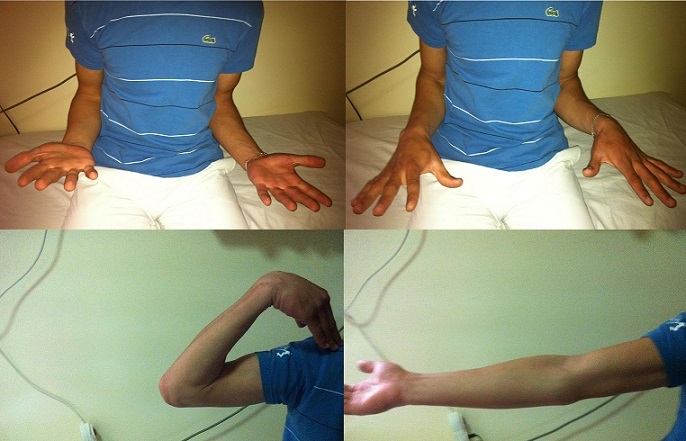
Résultat clinique à 2 ans

Une mobilité complète du coude avec absence de douleur a été obtenue après le troisième mois postopératoire. Avec un recul de 2 ans, le coude est stable mobile indolore (Figure 4).

## Discussion

L'ostéochondrite disséquante du capitellum (OCD) se définit comme une zone localisée de modification vasculaire atteignant l'os sous-chondral. En l'absence de cicatrisation, l'os et son couvercle cartilagineux vont se séparer de l'os adjacent créant un defect osseux et un corps étranger libre intra-articulaire [4].

L’étiologie de l'ostéochondrite disséquante reste inconnue. Historiquement il y a eu de nombreuses hypothèses physiopathologiques [4]. Enneking a proposé une théorie vasculaire liée à la fragilité de la vascularisation terminale de la zone épiphysaire. Un traumatisme peut interrompre cette vascularisation et celle des centres secondaires. Chez l'adulte, les traumatismes répétés peuvent entraîner des fractures de fatigue et un traumatisme vasculaire pouvant expliquer la formation d'une zone dévascularisée.

La clinique n'est pas spécifique. La sensation d'accrochage est possible dans les mouvements de flexion-extension. Le blocage vrai est rare et est habituellement le témoin d'une lésion ostéochondrale libérée dans l'articulation.

Baumgarten et coll [5] ont publié en 1998 une classification en 5 stades basés sur l'aspect arthroscopique des lésions: Au stade 1, il existe un ramollissement du cartilage; Au stade 2, une fissuration superficielle du cartilage; Au stade 3, exposition de l'os, le cartilage restant attaché; Au stade 4, le clapet osseux est mobile mais en place; Au stade 5, le fragment est libre dans la cavité articulaire.

L'arthroscanner, voire plus récemment l'arthro-IRM permettent de répondre à trois questions importantes: Quelle est la vitalité du fragment osseux?, Quelle est la stabilité du fragment ostéochondral dans sa « niche »?, Quel est l’état du cartilage?

Le traitement des lésions d'ostéochondrite disséquante étendue du capitellum reste difficile. La majorité des patients sont de jeunes athlètes et la qualité du résultat, surtout en termes de reprise de l'activité sportive de haut niveau reste controversée.

De nombreuses techniques chirurgicales ont été décrites: Le curetage simple de la lésion est une technique peu invasive, surtout quand il est réalisé par arthroscopie [6]. Beaucoup d'auteurs s'accordent à dire que ce traitement reste insuffisant pour des lésions étendues (plus de 20mm) avec un risque de remaniement arthrosique; Différentes techniques d'ostéosynthèse du fragment exclu ont également été rapportées [4] utilisant des broches de Kirchner, des broches résorbables ou des vis enfouies; Des techniques par greffe des cellules cartilagineuses autologue en culture ont été décrites avec un bon résultat à court terme, mais avec un recul encore insuffisant pour estimer la pérennité du résultat [7].

La greffe ostéochondrale: la mosaïcoplastie reste une technique difficile à réaliser au niveau du coude en raison de l'exiguïté de l'articulation huméro radiale. Certains auteurs ont utilisé un greffon ostéocartilagineux prélevé sur une côte plus facile à fixer [8].

Vu la taille de la lésion dans notre cas, nous avons réalisé la technique de microfractures du fond de la niche telle qu'elle a été décrite par Steadman [9]. Cet auteur a repris l'idée des perforations de Pridie, estimant qu'il peut ainsi entraîner la formation d'un caillot de régénération sans provoquer de nécrose osseuse, tout en réalisant une surface irrégulière permettant l'accrochage de ce caillot.

L’évolution arthrosique de la lésion est diversement appréciée dans la littérature, mais selon Bauer et al sur une série de 31 patients, 61% présentaient des lésions arthrosiques à 15 ans de recul [10].

## Conclusion

Le curetage simple de la lésion dans le traitement de l'ostéochondrite disséquante du capitellum chez l'adolescent est une solution chirurgicale raisonnable, offrant un résultat fonctionnel acceptable, sans pour autant prévenir une évolution arthrosique à long terme.
